# The uncanny valley effect and immune activation in virtual reality

**DOI:** 10.1038/s41598-025-15579-4

**Published:** 2025-08-19

**Authors:** Esther K. Diekhof, Svenja Kastner, Donna Deinert, Monika Foerster, Frank Steinicke

**Affiliations:** 1 Department of Biology, Neuroendocrinology and Human Biology Unit, Institute for Animal Cell- and Systems Biology, Faculty of Mathematics, Informatics and Natural Sciences, Universität Hamburg, Hamburg, Germany; 2 Department of Informatics, Human-Computer Interaction, Faculty of Mathematics, Informatics and Natural Sciences, Universität Hamburg, Hamburg, Germany

**Keywords:** Uncanny valley effect, Behavioral immune system, Error management theory, Psychoneuroimmunology, Human behaviour, Computer science

## Abstract

The uncanny valley effect describes a phenomenon where humanoid, almost lifelike virtual agents evoke feelings of discomfort in the observers. The *Pathogen Avoidance Hypothesis* proposes that these feelings are based on a cognitive mechanism that originally evolved to motivate pathogen avoidance. Slight imperfections in virtual agents are thereby misconceived as cues of communicable diseases that elicit disgust and avoidance behavior. Whether uncanny entities also increase mucosal immune responses, particularly when immediate avoidance is impossible, currently remains elusive. The present study examined the link between the uncanny valley and mucosal immune responses by comparing changes in the salivary antibody secretory immunoglobulin A (sIgA). For this, we developed three sets of virtual agents (cartoon, uncanny, and realistic agents) that differed in their human-likeness and uncanniness. Agents were encountered in a social virtual reality (VR) task that required close approach, making avoidance impossible. We found that only the set of uncanny agents who showed slight deviations from normal human appearance, yet were still considered as more human-like than the cartoon agents, evoked a significant increase in sIgA release during social interactions in VR. This conforms to error-management theories suggesting that perceptual systems are biased towards false positive responses to potential contagion cues, reducing the risk of missing actual health threats. Our findings further indicate that presence in virtual reality was likely essential for effective immune activation by uncanny agents, as increased sIgA levels correlated with deeper involvement in the virtual environment. Moreover, the immune response may have been automatically triggered by perceived threats from these agents, as conscious evaluations of interoceptive awareness, state anxiety, and eeriness during VR did not correlate with higher antibody release. Altogether, these data show that perception of uncanniness in VR evokes mucosal immune responses resembling those related to real contagion threats.

**Supplementary Information:**

The online version contains supplementary material available at 10.1038/s41598-025-15579-4.

## Introduction

In 1970, Mori first described the *uncanny valley phenomenon*, where entities such as robots or virtual agents, that appear nearly human, evoke discomfort due to slight, noticeable differences from normal human appearance^1^. Different theories have tried to explain the *uncanny valley effect* focusing on either the evolutionary, perceptual, or cognitive aspects of processing (see Wang et al.^2^ for overview). Among these, the *Pathogen Avoidance hypothesis* posits that uncanny faces may signal a higher risk of communicable diseases, activating evolved responses for pathogen avoidance^3,4^. Specifically, as realistic human-like entities often exhibit subtle deviations from expected human appearance, these deviations may become salient indicators of potential illness^5–7^. Although, the hypothesis has never been directly tested in virtual reality (VR), there is already some evidence indirectly supporting this assumption. Prior studies that used human stimuli (e.g., photos or videos of humans) have already shown that people with morphological divergences, such as skin lesions like acne or eczema, facial or bodily deviations such as cleft palates, large birth marks, extreme obesity, or physical disabilities are often misperceived as potential carriers of disease. This can evoke increased disgust, avoidance, and contamination concerns in the observers despite the absence of a real contagion threat^8–10^. In addition, humans are even sensitive to very subtle sickness cues such as pale lips, redder eyes, patchy skin or changes in gait patterns as shown in studies that induced a transient inflammatory state by controlled endotoxin challenge^11,12^. Since humans cannot directly perceive pathogens, they have to rely on indirect sensory and social cues that correlate with increased infection risk, but which are not always reliable indicators^13–15^. This tendency towards false positive categorization of potential pathogens thereby seems plausible from an evolutionary perspective and also conforms with error-management theories of threat detection under uncertainty^16,17^. Pathogens exerted strong selection pressures on humans. The consequences of failing to identify an actual contagion risk were significantly more severe than the consequences of mistakenly identifying a non-existent threat, thus promoting a bias towards false positives in the recognition of disease indicators. This led to various adaptations that not only enable adequate reactions to acute pathogen infestation, but also aid the detection of sickness indicators^13,18–21^.

Typical cues of infection or signals of a heightened contagion risk thereby activate a response repertoire that protects against pathogen infestation. This ‘Behavioral Immune System’ (BIS) involves affective and behavioral strategies such as disgust or avoidance of potential pathogens^15,19^. Additionally, activation of the BIS can also trigger defensive immune responses at mucosal borders in situations of almost certain contagion, e.g., when sneeze aerosols are difficult to avoid^22–25^ and these mucosal immune responses may further interact with affective responses like disgust sensitivity^26^. Interestingly, Keller et al.^7^ not only observed that sneezing human-like agents at a virtual bus stop evoked this proactive mucosal immune response, i.e., increased release of salivary secretory immunoglobin A (sIgA), but also found a similar rise in sIgA during social interactions with virtual agents, who did not sneeze. Secretory IgA is an important component of the first-line of immune defense at mucosal barriers, especially against respiratory viruses. It thereby protects the epithelium by mechanisms such as immune exclusion and intracellular neutralization^27,28^. Our observation of an increased sIgA release during interaction with virtual agents raises intriguing questions about the mechanisms by which virtual agents trigger immune responses despite lacking the disease indicators that are commonly found to elicit increased sIgA release. It thus seems reasonable to assume that the visual deviations from typical human features in virtual agents might have been the source of this unexpected immune response. In fact, upon closer inspection we found that the human-like agents used by Keller et al.^7^ carried some conspicuous abnormalities, including (1) an unnaturally idle posture, (2) the tendency of becoming cross-eyed upon being approached, (3) a smile that did not reach the eyes, and (4) an awkwardly twisted upper body, which became particularly evident when the agents were closely approached. These abnormalities could have thus been easily misconceived as disease indicators, as suggested by the *Pathogen Avoidance Hypothesis*^2–4^. Since the task required participants to remain close to the agents until a smile was incited (which occurred after 15s on average), immediate avoidance was made impossible, which could have provoked the immunological tier of the BIS as protection against the potential contagion threat. Yet, the pathogen avoidance theory was only one possible explanation for this observation. Most of the data from Keller et al.^7^ were collected during the COVID-19 pandemic, when mask mandates at bus stops were still in effect. It was therefore equally likely that the scenario of a virtual bus stop with unmasked agents represented a potential contagion threat in itself, also given the general knowledge regarding pre- or asymptomatic viral transmission risk. This would also be in line with previous observations from the pandemic that suggested that disagreeable strangers may be considered a potential contagion threat in close social encounters even when seeming otherwise healthy^29^. In turn, the pandemic high-risk environment could have similarly been the trigger of defensive immune responses in close social interactions at a virtual bus stop regardless of typical sickness signs. However, it is also possible that the ongoing pandemic may have had no amplifying effect on the mucosal immune response at all. This is because acquired knowledge about the risk of asymptomatic transmission strongly relies on higher-order cognitive processing and thus may not effectively trigger an automatic defense mechanism that responds to the detection of typical sickness indicators, which are however absent in asymptomatic disease carriers (see^30^ for critical review). In that way, it seems probable that the increased mucosal immune response to agents carrying uncanny features will still be found in the present study.

Based on these insights, the current project investigated in further detail whether the uncanniness of virtual human-like agents was sufficient to evoke a proactive mucosal immune response, even outside of the pandemic high-risk context (Note: In May 2023, the World Health Organization (WHO) declared an end to the global Public Health Emergency for COVID-19.). To test this, we employed a between-subjects design where participants engaged in the *Make-All-Agents-Smile-Task* (MAAST; introduced by Keller et al.^7^ that involved a social interaction with one of three categories of virtual agents that varied along the dimensions of human-likeness and eeriness. These comprised (1) a set of 10 cartoon agents (low human-likeness, low eeriness), (2) the 10 uncanny agents from Keller et al.^7^ who carried several abnormal features (high human-likeness, high eeriness), and (3) a set of 10 improved realistic agents without the conspicuous abnormalities of the uncanny agents (high human-likeness, low eeriness). In each of the three test groups, the task goal was to make all agents of the respective category, who were standing at a bus stop, smile. For achieving this, individual agents had to be closely approached and eye contact had to be established. Then, after a short waiting period (time to smile averaged at about 15 s) the agent smiled and the participant could move on to the next agent. Participants provided saliva samples before and after the MAAST to measure changes in the sIgA secretion, and evaluated their feelings about the agents and their general VR experience. We hypothesized that the sIgA secretion would significantly rise during interactions with the uncanny agents, who may be misconceived as a contagion threat, but not when interacting with the other two agent categories. In addition, we expected that an increased presence and especially involvement in VR would correlate with higher sIgA increases in this context, as already observed by Keller et al.^7^. Finally, we predicted that the perception of uncanniness, state anxiety, and negative interoceptive feelings would positively correlate with sIgA changes (see also preregistered study protocol by Diekhof, Kastner, Löding, and Steinecke at https://osf.io/7uq6r/).

## Results

### Subjects

We tested 74 healthy persons, of whom 8 had to be excluded from the analysis for various reasons: Two provided insufficient material for sIgA analysis, two became nauseous and/or dizzy during VR (indicators of cybersickness), another two showed very high sIgA baseline values that exceeded the sample mean by more than two standard deviations, while the remaining two did not undergo the VR protocol due to technical problems. As a result, 66 participants were included in the final analysis (realistic group: *n* = 20, age = 24.4 ± 4.4 years, 13 women; uncanny group: *n* = 23, age = 23.6 ± 3.3 years, 14 women; cartoon group: *n* = 23, age = 23.3 ± 3.6 years, 16 women).

**Table 1:** Descriptive statistics and statistical comparison of trait and state measures.

	Realistic group		Uncanny group		Cartoon group		Group differences
	Mean	SD	Mean	SD	Mean	SD	p-value
Trait questionnaires							
Disgust	31.10	10.65	29.04	11.87	32.52	8.74	.630
Contamination Disgust	5.90	3.43	5.35	3.52	6.26	3.66	.598
Core Disgust	25.20	8.01	23.70	9.46	26.26	6.92	.715
Vulnerability To Disease	50.05	13.71	46.00	9.75	48.83	11.95	.581
Germ Aversion	28.75	7.44	27.87	6.33	29.22	8.27	.881
Perceived Infectability	21.30	7.79	18.13	5.02	19.61	5.58	.254
State questionnaires							
STAI (state anxiety)	16.20	8.00	17.00	8.80	16.22	5.73	.897
IPQ – General Presence	4.40	1.05	3.57	1.67	4.00	1.51	.299
IPQ – Involvement	3.48	1.07	3.42	1.60	3.52	1.38	.924
IPQ – Experienced Realism	2.44	1.08	1.84	1.32	1.90	1.24	.190
IPQ – Spatial Presence	3.57	0.74	3.17	1.03	3.13	0.94	.175
SSQ (Cybersickness)	6.75	6.38	6.35	4.98	6.35	4.44	.957
Scale of Uncanny Feelings	11.00	10.72	9.74	10.04	9.09	9.19	.855
Somatic Feelings Questionnaire	2.95	3.47	3.61	4.00	3.70	4.13	.702

Differences were assessed with the Kruskal-Wallis-test (*p* < .05, two-tailed).

The three groups did not differ in age (*p* = .776), nor in any of the trait measures of disgust or disease vulnerability or the state measures such as cyber sickness and anxiety (see Table 1 for a complete list of the comparisons of trait and state scores).

**Fig. 1:**
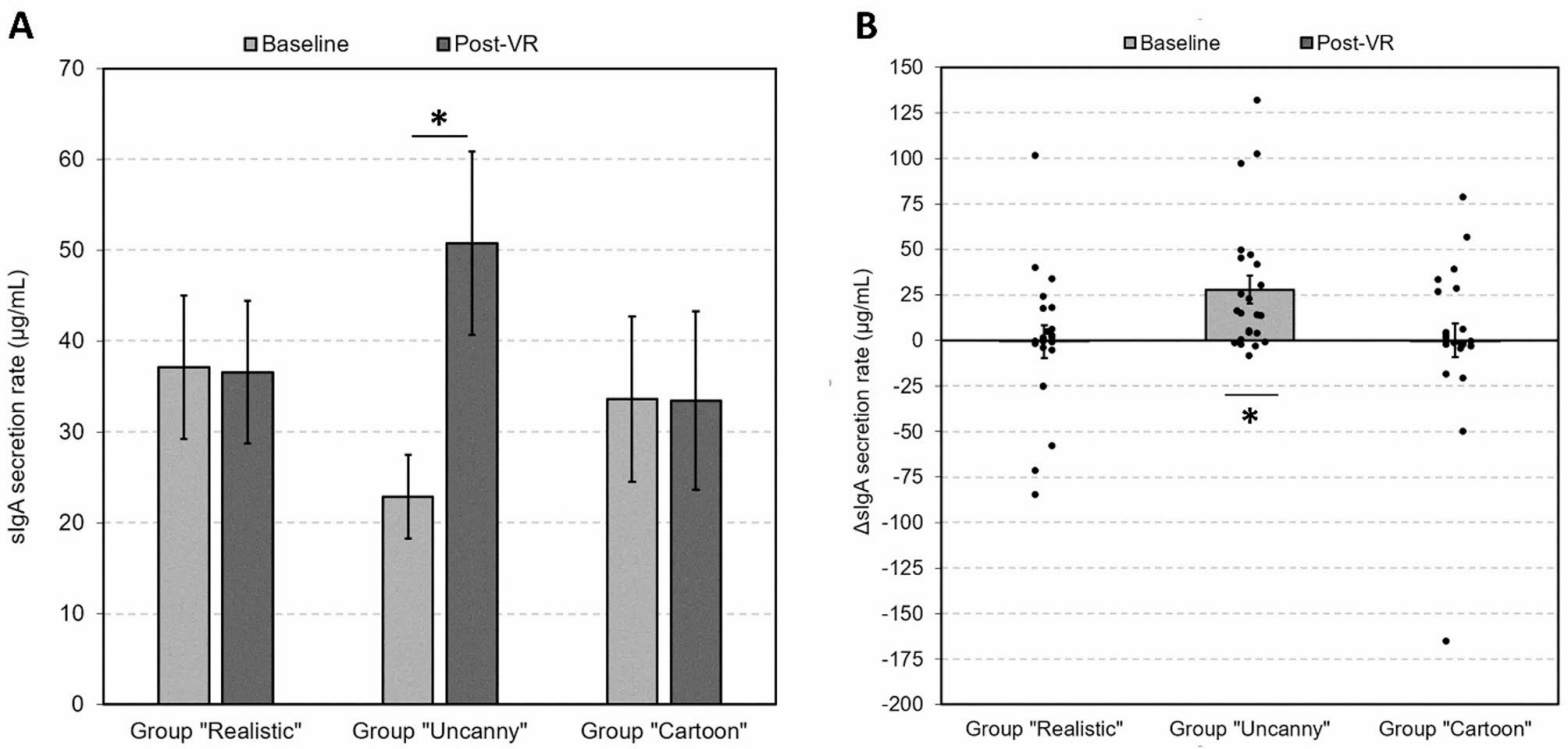
SIgA-changes during VR in the different agent groups. (A) Only in the group, who interacted with uncanny agents, we found a significant increase in sIgA from baseline to post-VR, while sIgA remained unaltered in the other two groups (*p* < .05). (B) The ΔsIgA significantly differed from zero only in the uncanny group (*p* < .05, two-tailed). Both figure parts display means and standard errors of the mean. Significant differences (*p* < .05, two-tailed) are marked with an asterisk. Figure 1B also contains individual data points.

### sIgA change during VR

The 2 × 3 GLM included the factors “sample” (baseline, post-VR) and “group” (realistic, uncanny, cartoon) to examine their interactive effect on sIgA secretion rate. We neither found a significant main effect of “sample” (F(1,63) = 3.22, *p* = .078, partial η^2^ = 0.049) nor one of “group” (F(2,63) = 0.07, *p* = .933, partial η^2^ = 0.002). However, the interaction between the two factors was significant (F(2,63) = 3.55, *p* = .035, partial η^2^ = 0.101). Post-hoc pairwise comparisons with the Mann-Whitney-U-test showed that the increase in sIgA secretion from baseline to post-VR (ΔsIgA) was significantly higher in the participants of the uncanny than of the realistic (U = 141, Z=-2.17, *p*=-.030, two-tailed) or the cartoon group (U = 158, Z = -2.34, *p* = .019, two-tailed) (Fig. 1A; Table 2). Further, only in the uncanny group, ΔsIgA secretion rate differed significantly from zero (Z = -3.41, *p* < .001, two-tailed), while there was no difference in the realistic (Z = -0.26, *p* = .794, two-tailed) and the cartoon group (Z = -0.34, *p* = .738, two-tailed) (Fig. 1B).

**Table 2:** Descriptive statistics and statistical comparison of sIgA secretion rate.

SIgA secretion rate (µg/mL)	Realistic group		Uncanny group		Cartoon group		Group differences
	Mean	SD	Mean	SD	Mean	SD	p-value
Baseline	37.12	35.12	22.91	22.14	33.61	43.67	.453
Post-VR	36.58	34.97	50.75	48.37	33.49	46.95	.309
Delta (Post-VR – Baseline)	**-0.55**	**40.15**	**27.83**	**37.29**	**-0.12**	**44.85**	.031*

Differences were assessed with the Kruskal-Wallis-test (*p* < .05, two-tailed). Results of pairwise post-hoc comparisons can be found in the text. Significant differences are marked with an asterisk and highlighted in bold.

### ΔsIgA and presence in VR

**Fig. 2:**
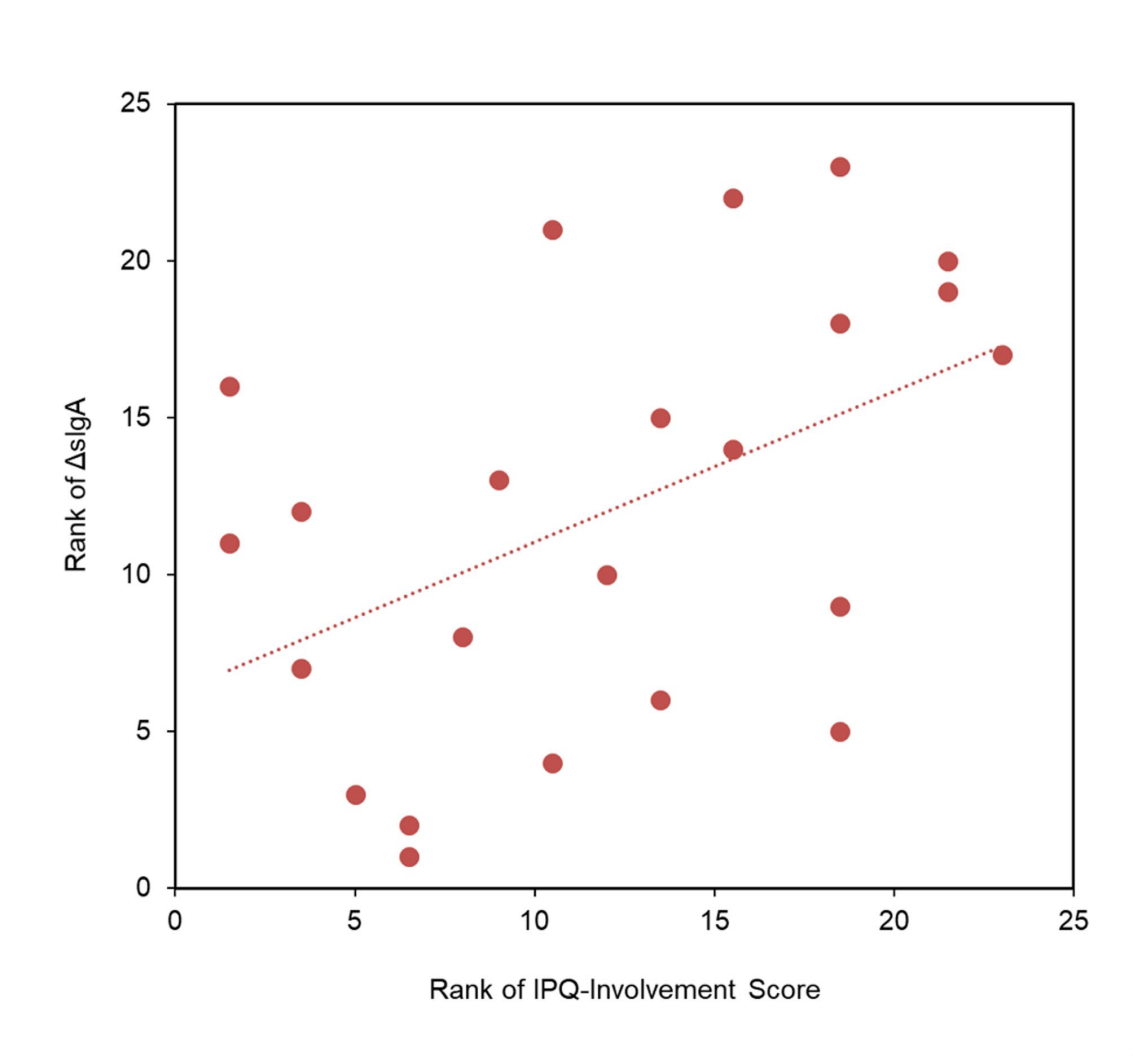
Positive correlation between sIgA increase during VR and IPQ-Involvement in the uncanny group. Figure displays rank values of non-parametric data. The significant positive correlation (Rho = 0.478, *p* = .021) is indicated by the dotted line.

The correlation of ΔsIgA and the sub-scores of the IPQ revealed a significant positive correlation with IPQ *Involvement* (Rho = 0.478, *p* = .021, two-tailed) (Fig. 2), and a trend-wise positive correlation with IPQ *Experienced Realism* (Rho = 0.372, *p* = .080, two-tailed) in the uncanny group, yet not in the other two groups. The remaining IPQ scales were uncorrelated with ΔsIgA (Table 3).

### ΔsIgA, interoceptive feelings and state anxiety

When exploring the correlations between ΔsIgA, interoceptive feelings and state anxiety, we found no evidence for a significant association between the physiological measure and state feelings, neither when the data were separated by group nor when assessing the complete sample (Table 3).

### ΔsIgA and perceived uncanniness

We also assessed the correlation between the eeriness ratings of the uncanny agents (sum score) and ΔsIgA in each of the three groups and examined whether the individual score of the Scale of Uncanny Feelings was somehow related to ΔsIgA. Yet, no significant correlations emerged (Table 3).

**Table 3:** Spearman rank correlations of ΔsIgA with questionnaires scores and agent ratings.

	Realistic group		Uncanny group		Cartoon group		All participants	
	Rho	p	Rho	p	Rho	p	Rho	p
Questionnaire scores								
IPQ – Presence	.074	.756	.125	.569	− .038	.862	< .001	.997
IPQ – Involvement	− .096	.688	**.478**	.021*	− .161	.462	.102	.416
IPQ – Experienced Realism	.122	.610	**.372**	**.080** ^**+**^	− .305	.156	− .019	.881
IPQ – Spatial Presence	.189	.426	.167	.446	− .053	.812	.041	.745
SSQ (cybersickness)	.043	.857	− .056	.801	− .106	.630	− .021	.868
Scale of Uncanny Feelings	.225	.339	− .129	.559	− .110	.619	− .018	.884
Somatic Feelings Questionnaire	.327	.160	− .155	.479	.069	.753	.105	.403
STAI (state anxiety)	.010	.967	− .157	.475	.129	.558	− .025	.843
Mean agent ratings								
Human-likeness (realistic agents)	− .044	.855	.127	.565	.161	.464	.089	.479
Attractiveness (realistic agents)	.041	.865	− .015	.946	− .015	.945	.053	.671
Eeriness (realistic agents)	.114	.633	− .271	.212	− .199	.363	− .079	.528
Human-likeness (uncanny agents)	.026	.915	.236	.278	.261	.229	.115	.357
Attractiveness (uncanny agents)	.233	.324	− .049	.824	− .105	.634	.075	.551
Eeriness (uncanny agents)	.050	.835	− .142	.518	− .170	.438	− .028	.824
Human-likeness (cartoon agents)	.200	.397	− .110	.616	.158	.471	− .072	.563
Attractiveness (cartoon agents)	.353	.127	− .211	.335	− .136	.536	− .025	.845
Eeriness (cartoon agents)	− .185	.434	.330	.124	− .123	.577	− .023	.853

The significant correlation (*p* < .05, two-tailed) is written in bold and marked with an asterisk. The trend-wise correlation (*p* < .10, two-tailed) is written in bold and marked by a cross.

### Group differences in agent ratings

Finally, we also explored the differences in the rating dimensions. For this, we used a GLM with the two within-subject factors “agent category” (cartoon, uncanny, realistic) and “rating dimension “(human-likeness, attractiveness, eeriness), as well as the between-subjects factor “group” (cartoon, uncanny, realistic). The between-subjects factor was included to account for the restricted VR experience with only one of the three agent categories in each group, i.e., the “own-group effect”, that might have influenced the subsequent ratings of the short video sequences of the different agents. The following post-hoc comparisons focus on the eeriness ratings as this aspect was central to the current project. The complete overview of the results from the GLM and the associated descriptive statistics can be found in Tables 4 and 5 as well as in the Supplementary data.

**Table 4:** Results of the repeated measures 3 × 3 × 3 GLM of agent category, rating dimension and group.

Main effect or interaction	df	F	Sig.	Partial η^2^
Agent category	**1.41**,** 88.78**	268.48*	**< .001**	**0.81**
Agent category x group	**2.82**,** 88.78**	4.27*	**.008**	**0.12**
Rating dimension	**1.59**,** 100.01**	32.52*	**< .001**	**0.34**
Rating dimension x group	3.18, 100.01	1.94	.125	0.06
Agent category x rating dimension	**1.88**,** 118.14**	42.84*	**< .001**	**0.41**
Agent category x rating dimension x group	**3.75**,** 118.14**	2.83*	**.031**	**0.08**
Group	2, 63	0.81	.452	0.03

Df are corrected by Greenhouse-Geisser as data failed to match the sphericity criterion.

We observed that “agent category” significantly affected the rating dimensions (interaction of “agent category” x “rating dimension”: F(1.88, 118.14) = 42.84, *p* < .001, partial η^2^ = 0.041). Pairwise comparisons with the Wilcoxon test showed that across the complete sample the uncanny agents were rated as more eerie than the realistic (Z=-4.22, *p* < .001) and the cartoon agents (Z=-2.34, *p* = .019). The cartoon agents were however not considered as more eerie than the realistic agents or vice versa (Z=-0.51, *p* = .609). In terms of the dimensions of human-likeness and attractiveness we also found the expected rating hierarchy of “realistic > uncanny > cartoon agents” (see column “All” in Table 5 for mean values and Table S1 for results of pairwise comparisons).

**Table 5:** Descriptive statistics of rating dimensions for each agent category and group (sum scores of the ratings of the 10 agents of each category).

	Realistic group		Uncanny group		Cartoon group		All		Group differences
	Mean	SD	Mean	SD	Mean	SD	Mean	SD	p-value
Human-likeness (realistic agents)	52.65	14.29	58.96	22.54	62.83	14.72	58.39	17.95	.092
Attractiveness (realistic agents)	36.10	14.61	38.00	21.96	34.17	15.09	36.09	17.47	.568
Eeriness (realistic agents)	25.55	20.31	23.61	17.36	24.09	16.68	24.36	17.81	.972
Human-likeness (uncanny agents)	56.45	19.77	50.91	21.98	58.48	17.79	55.23	19.89	.400
Attractiveness (uncanny agents)	34.85	15.08	35.61	18.27	26.70	13.70	32.27	16.12	.068
Eeriness (uncanny agents)	28.45	26.22	30.78	16.90	33.61	20.03	31.06	20.93	.402
Human-likeness (cartoon agents)	**8.60**	**6.45**	**10.22**	**15.03**	**30.91**	**16.94**	16.94	17.10	< .001*
Attractiveness (cartoon agents)	6.40	9.08	8.17	14.74	15.00	13.92	10.02	13.31	.061
Eeriness (cartoon agents)	30.20	30.07	21.26	25.50	21.74	20.04	24.14	25.23	.634

Differences were assessed with the Kruskal-Wallis-test (*p* < .05, two-tailed). The significant difference is marked with an asterisk and highlighted in bold.

**Fig. 3:**
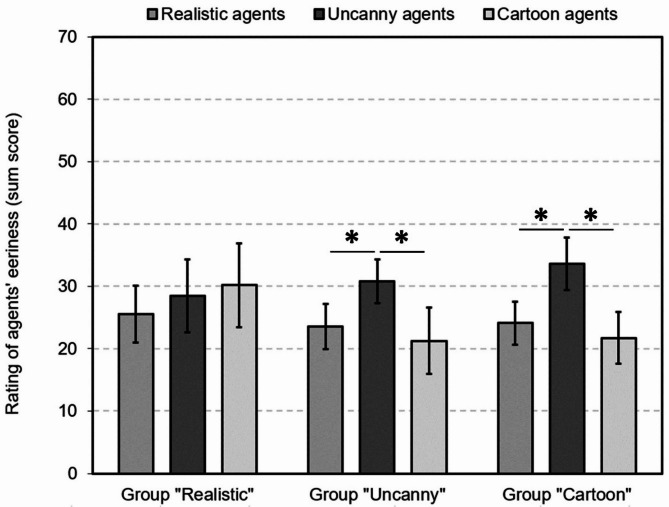
Ratings of agents’ eeriness by the different groups. Figure displays mean values and standard errors of the mean. Significant differences are indicated by an asterisk (*p* < .05, two-tailed).

When examining the significant three-way interaction of “agent category” x “rating dimension” x “group” (F(3.75, 118.14) = 2.83, *p* = .031, partial η^2^ = 0.08), post-hoc Wilcoxon-tests separated by group showed that the uncanny agents were perceived as significantly more uncanny in comparison to realistic agents by the participants of the uncanny group (Z=-3.13, *p* = .002) and of the cartoon group (Z=-3.61, *p* < .001). This was also the case for the comparison of the uncanny agents with the cartoon agents (uncanny group: Z=-1.97, *p* = .049; cartoon group: Z=-2.74, *p* = .006). Only when separately assessing the realistic group, neither the uncanny agents were perceived as more eerie than the realistic agents (Z=-0.58, *p* = .559) nor the cartoon agents (Z=-0.32, *p* = .747). Instead, the agent ratings were similar for all agent categories (Table 5; see also Fig. 3).

## Discussion

Pathogens represented a significant selection pressure during human evolution. This not only led to the development of a physiological immune system, but also endorsed a cognitive mechanism – the BIS – by which disease cues and contagion predictors can be identified even before pathogen contact. Consequently, the BIS evokes feelings of disgust and triggers avoidance behavior upon detection of disease indicators^14,19^ and can activate immune responses, in case contagion cannot be easily evaded^24,25,31^. Notably, the BIS does not only respond to typical symptoms of communicable diseases, but can also be activated by deviations from normal human appearance, such as skin rashes, paleness or facial lesions, that are often misconceived as contagion indicators^8–10^. In the present study, we directly assessed to what extent variations in the eeriness of human-like virtual agents, that are caused by slight deviations from a natural human look, affect the mucosal immune response in close social interactions in VR. We found that interactions with human-like uncanny agents evoked a significant increase in sIgA release in saliva, which was not observed during close social interactions with either cartoon-like agents or an improved category of realistic human-like agents. The sIgA rise to uncanny agents further positively correlated with the experienced involvement and realism in/of the VE, implying a supporting influence of presence on immune activation by uncanniness. Notably, other aspects of presence, interoceptive awareness, and state anxiety did not correlate with the degree of sIgA release. This suggests that the observed immune response may have been automatically engaged and thus seems to be widely independent of conscious evaluations of the potential contagion threat. It may thus act as an effective threat detector and defense mechanism, also in the virtual world.

Our results support the *Pathogen Avoidance Hypothesis* as explanation of the uncanny phenomenon^2–4^. According to this theory, humans show increased sensitivity to abnormalities in human-like entities, because their similarity in physical and behavioral attributes suggests genetic relatedness, which is associated with a higher risk of disease transmission^32^. We found that only the human-like uncanny agents triggered an activation of the immunological tier of the BIS, suggesting that their slightly deviant features may have been indeed misconceived as a contagion threat. This also conforms with other observations that found deviations from typical human appearance, such as facial lesions, rashes or scars, to falsely activate the BIS^8–10^. With regard to the results from our previous VR-study that was conducted during the COVID-19 pandemic^7^we can thus rule out, that the general contagion threat of the pandemic, which rendered all social interactions between humans as potentially risky, was a major driving force of the increased immune activation in VR. This would also conform with the notion of the BIS as an evolutionary conserved mechanism that evolved in response to typical disease threats, yet not to theoretical concepts such as those concerning asymptomatic transmission^30^. Our results thus also extend previous findings that showed a relation between eeriness perception and the cognitive-behavioral tier of the BIS, i.e., disgust-sensitivity^33^ by demonstrating a direct association of uncanniness with the first-line-of-defense immune response at the oral mucosae, which suggests that uncanniness falsely triggered an automatic disease detection mechanism.

Increased realism in human-likeness has been found to lower the tolerance for abnormalities from objective realism^34^. The eeriness of virtual agents may thereby be particularly associated with a lack of objective realism in the eye and mouth region^35^. These regions are important for face and emotion recognition in social interactions and may thus be prioritized during initial perception^36,37^. Our set of uncanny agents exhibited deviations in both the eye and the mouth region. In contrast, the realistic agents did not carry these aberrant features, but were carefully improved to exhibit a more consistent and welcoming appearance overall. This not only included a more natural smile and normal eye movements, but also a generally more realistic movement pattern, which had previously been shown to reduce the uncanniness of human-like entities^38^. In contrast, the cartoon agents’ face looked rather plain, with minimal resemblance to a real human face, i.e., black dots as eyes and a crooked line that represented the smiling mouth. Consistent with this, the uncanny agents were rated as significantly more eerie than agents from the other two categories (when considering the whole sample and the uncanny and cartoon group separately). Surprisingly, the general score of the *Uncanny Feelings Scale* did not differ between the groups, and the groups also showed no difference in aversive somatic feelings, while interacting with the different agent categories. We would thus assume that the BIS may have responded automatically to the perceived contagion threat of the uncanny agents, which apparently did not necessitate the conscious experience of an eerie or aversive somatic feeling. This would also explain, why both the realistic and the cartoon agents did not trigger a consistent increase in antibody release on the group level, despite the similarity in the subsequent psychological evaluations of subjective feelings.

While our finding fits with the *Pathogen Avoidance Hypothesis*, we cannot ignore other explanations of the uncanny phenomenon. The *Mortality Salience hypothesis*^3,32^ posits that uncanny agents resemble dead individuals and may thus be a reminder of one’s own mortality, evoking a deep-rooted fear of death which reflexively triggers mechanisms of threat avoidance. Even though we cannot rule out general fear responses during encounters with the uncanny agents, our data provide no such indication. For one thing, the participants from the different groups had comparable scores on the STAI questionnaire. For another, a general fear response normally does not involve an immune activation. Approaching or touching corpses can however activate the BIS^39^. Thus, increased sIgA release in our particular setting more likely reflects a defensive immune response triggered by the perceived contagion potential than a fearful reaction to an abstract death threat.

The *Evolutionary Aesthetics hypothesis*^3,40^ posits that humans are highly sensitive to facial aesthetics that signal physical health, fertility and genetic fitness. Uncanny entities partly fail to meet these aesthetic standards, rendering them unattractive, which contributes to the eerie feeling experienced. In our study, the attractiveness ratings of the realistic and uncanny agents did not differ from those of the uncanny group, arguing against this assumption. Further, the *Evolutionary Aesthetics hypothesis* mainly focusses on the ultimate level of selection pressures and genetic fitness, rather ignoring the proximate link between unaesthetic facial features and current health (e.g., unusual skin texture or coloration may indicate acute inflammation^41^. We assume, that the ultimate motives proposed in the *Evolutionary Aesthetics hypothesis* could have contributed to the development of the proximate mechanisms described in the *Pathogen Avoidance Hypothesis*, which would make the latter theory the most fitting to account for the present experimental results, while not ruling out the former.

We will not discuss the remaining cognitive-based theories here, i.e., the *Violation of Expectation*, *Categorial Uncertainty*, and *Mind Perception Hypothesis*^2^ as they provide no explanation for the increased immune activation in the uncanny group and further cannot be adequately tested with our experimental design.

The present data show that the proactive immune activation occurred in the situation, in which immediate avoidance of the presumed source of increased pathogen load, i.e., the uncanny agents, was impossible. The MAAST required that participants closely approached the agents and stayed near them for some time. To completely characterize our specific finding, the term *Pathogen Defense* mechanism would thus be more appropriate than *Pathogen Avoidance*. As already shown by Keller et al.^7^ the extent of sIgA increase was thereby associated with interindividual differences in the involvement in the VE and also the experienced realism. This further adds to the assumption that the subjective feeling of of being present in VR combined with the ineluctability of the uncanny agents in a close social interaction in VR successfully mimicked a real-life, potentially contagious situation that triggered the increased mucosal immune activation. Previous research on the association between presence and emotional responses already implied that aversive stimuli that increase alertness lead to greater perceived physical and mental presence in VR^42^. It would be interesting to further assess, whether a similar sIgA increase can be observed when participants are given the change of immediate escape. In that case, we would expect no significant change, if not even a drop, in sIgA.

Finally, our observation also conforms with error-management theories of decisions under uncertainty. Since contagion indicators are not perfectly diagnostic, misinterpretations may be biased towards inferring threat in order to reduce individual risk^16^. Acting in favor of false positive responding would thereby be less costly than falsely recognizing an actual, potentially damaging disease threat as safe^17,43^. We did not explicitly ask our participants for their evaluation of the uncanny agents’ health status. Still, the sIgA release during VR was increased when being exposed to the uncanny agent category, which probably mimicked a realistic health threat^24,25,31^. In that way, we can indeed infer that the uncanny agents may have been falsely classified as contagious, at least unconsciously, which was driven by biased perception in favor of a false positive response.

Yet, our study only assessed one aspect of the mucosal immune response (sIgA in saliva). This was done, because sIgA represents a reliable indicator of immune activation to contagion indicators that showed a robust increase across different intervention-modalities (i.e., after having watched videos with typical contagion indicators, while either sitting at a desk or lying in an MR-scanner, and following close social interactions with sneezing agents in VR) and over various exposure durations (from 2:40 min to ~ 24 min) as previously shown^7,24,25,31,44^. Nevertheless, future studies should consider a more comprehensive assessment of salivary immune markers that also includes inflammatory and other antimicrobial components (e.g., TNF-α, α-amylase, cytokines), which were found to be increased in other studies on disease-related disgust (e.g.^23,45–47^, although not as reliable as salivary sIgA.

In conclusion, our study showed that variations in the eeriness of human-like virtual agents, resulting from slight deviations from natural appearance, influenced the mucosal immune response during close social interactions in virtual reality. The increased release of sIgA in saliva in response to the uncanny agents may thereby reflect an effective threat detection and defense mechanism, that can be engaged even in virtual contexts. It requires a certain extent of involvement in the VE, yet may operate largely independent of conscious assessments of the actual contagion risk. In that way, it may automatically protect humans in close social interactions with conspecifics even when possible contagion signs are far from being reliable indicators of a disease.

## Materials and methods

### Participant recruitment

We recruited participants on university campus of the University of Hamburg, through online advertisements and via social media. We only invited healthy individuals to participate, who (a) indicated German as a native language, (b) were of legal age (at least 18 years old), but not older than 35 years, (d) were not smoking regularly, (e) had no hormonal, genetical, nor other chronical diseases, and (f) had not been vaccinated in the last two weeks. For determination of sample size, we conducted a power analysis with G*Power 3.1.9.4^48^ beforehand, which used the repeated-measures 2 × 3 ANOVA (sample X group) with “within-between interactions”, and used an expected effect size of f = 0.25, which translates to an η^2^ = 0.10 as a small effect, an α = 0.05, and a power of 95%. This resulted in a sample size of 66 persons. In the end we tested 74 participants, since we wanted to account for potential drop-out. Participants were randomly assigned to one of the three test groups, while trying to balance the sex ratio between groups. The procedure was approved by the local ethics committee (“*Ethikkommission der Ärztekammer Hamburg*”), and conformed with the Declaration of Helsinki. The participants gave written informed consent and received a financial reward of 20 Euros for completing the appointment.

### Procedure

Before scheduling an appointment for the study, each participant needed to complete an online questionnaire created with *LimeSurvey*, in which demographic and health-related data were collected. Participants also indicated their previous experience with a VR headset, the general tendency for cybersickness, as well as their trait disgust propensity (revised Disgust Scale [DSR])^49^ and the Perceived Vulnerability to Disease^50^ by answering the questions of the two validated test inventories (The content of the two inventories is described in more detail in the section “Questionnaires” below). All questionnaires were administered in German.

The actual VR experiment took place at the Institute of Animal Cell and Systems Biology (University of Hamburg, Germany). Participants were always tested between 12 p.m. and 5 p.m. to account for the circadian rhythm of sIgA^51^. Upon arrival, the participant first signed the consent from and was given general information on the experiment. Then he/she started with an initial saliva sample to get accustomed to the passive drool method. This sample was later discarded, as it simply represented a practice sample. Next, the participant watched a five-minute relaxation video showing videos of nature and landscape impressions accompanied by relaxing music to reduce potential stress in the unfamiliar test environment. Right after the relaxation video, he/she provided the second saliva sample, i.e., the baseline sample, while sampling time was recorded. Following the baseline sample, the participant answered some demographic questions (age, sex) and questions related to recent stress, consumption of legal substances such as caffeine or nicotine and other health-related aspects from the test day, which, as confounding variables, would have allowed later exclusion (e.g., if a participant indicated heightened stress level and use of antibiotics on the test day). Once finished, the participant was instructed to put on the VR headset. The investigator explained the controls and the task to the participant and started the tutorial of the MAAST. When the participant felt comfortable with the controls and finished the tutorial, the actual VR scenario was started and the time to completion of the MAAST was recoded. Directly following the VR scenario, the participant was asked to provide the third saliva sample, i.e., the post-VR sample, of which the sampling time was recorded. This last saliva sample was followed by a post-scenario questionnaire, which included several validated test inventories that were translated into German and are described in more detail below: (1) the Scale of Uncanny Feelings^5^(2) the Simulator Sickness questionnaire (SSQ)^52^(3) the Igroup Presence questionnaire (IPQ)^53^(4) the Somatic Feelings questionnaire^54^(5) ratings of the 30 agents regarding the dimensions of human-likeness, attractiveness, and uncanniness, and (6) the State Anxiety questionnaire (STAI)^55,58^. All questionnaires were used with permission (i.e., were freely available, given correct reference to the authors, or were obtained from university resources).

### Questionnaires

During recruitment, participants answered 17 items from the DSR^49^. Eight items contained true/false statements like “*I might be willing to try eating monkey meat under some circumstances.* “, for which participants indicated their agreement on a 5-point Likert-scale (from 0 = ‘*Strongly disagree*’ to 4 = ‘*Strongly agree*’). The rest of the items described disgust-eliciting situations such as: “*While you are walking through a tunnel under a railroad track*,* you smell urine.*”, which were rated from 0 = ‘*Not disgusting at all*’ to 4 = ‘*Extremely disgusting*’. The items were added up to two sub-scores [Contamination Disgust (5 items) and Core Disgust (12 items)] and the overall sum score of Disgust. Apart from that, participants evaluated their Perceived Vulnerability to Disease, by using a 15-item self-report instrument^50^. The 15 items included statements like “*In general*,* I am very susceptible to colds*,* flu*,* and other infectious diseases.*”, which the participants had to rate on a 7-point scale from 1 = ’*Strongly disagree*’ to 7 = ‘*Strongly agree*’. These items were combined into a sum score as well as the two sub-scores of Perceived Infectability (8 items) and Germ Aversion (7 items).

On the test day, another set of questionnaires was answered directly after VR. The Scale of Uncanny Feelings^5^ consisted of 16 items with statements such as ”*I feel creeped out.*” and ”*I have a feeling that I’m not in charge of my actions.*” to measure unsettling emotions that can be triggered by the experience of an uncanny valley effect. Items were rated on a 7-point scale, ranging from 0 = *’I do not agree at all*’ to 6 = ’*I completely agree*’. The SSQ assessed cybersickness in VR^56^. It contained 16 items that describe feelings such as general discomfort, fatigue, and nausea evoked by VR, which were answered on a 4-point Likert scale, ranging from 0 = *’Not at all* ’ to 3 = ’*Very much*’. The IPQ^53^ assessed the feeling of presence in VR by statements containing 14 items such as ”*I had a sense of seeing only pictures.*” and ”*I felt present in the virtual space.*”, which were evaluated with a 7-point Likert scale, ranging from 0 = ’*Does not apply at all* ’ to 6 = ’*Fully applies*’. It consisted of the IPQ-subscales of *General Presence* (1 item), *Spatial Presence* (mean of 5 items), *Involvement* (mean of 4 items), and *Experienced Realism* (mean of 4 items). The Somatic Feelings questionnaire^54^ retrospectively measured the subjective interoceptive feelings, such as physiological sensations, experienced during VR. We used the modified version from Keller et al.^25^ that contains 13 statements such as ”*I felt like I could vomit during the scenario.*” and ”*I felt unclean during the scenario*.”, which were evaluated on a 7-point Likert scale, ranging from 0 = *’Not at all* ’ to 6 = ’*Very much*’). Moreover, participants also rated their own agents as well as the agents from the other two categories regarding the dimensions of human-likeness, attractiveness and uncanniness^57^. For this, they watched 10-seconds video clips of each agent that ended with a smile. For rating of each video, we used an 11-point Likert scale, ranging from 0 = ’*Not at all*’ to 10 = ’*Totally*’), answering the three questions “*How human-like do you find this agent*?”, “*How attractive do you find this agent?*” and “*How eerie do you find this agent?*”. State anxiety elicited by the VR experience was measured by the STAI^58^. Participants evaluated 20 items such as ”*I feel down*” on a 4-point Likert scale, ranging from 0 = ’*Not at all*’ to 3 = ’*Very much*’.

### Virtual environment and agents

**Fig. 4:**
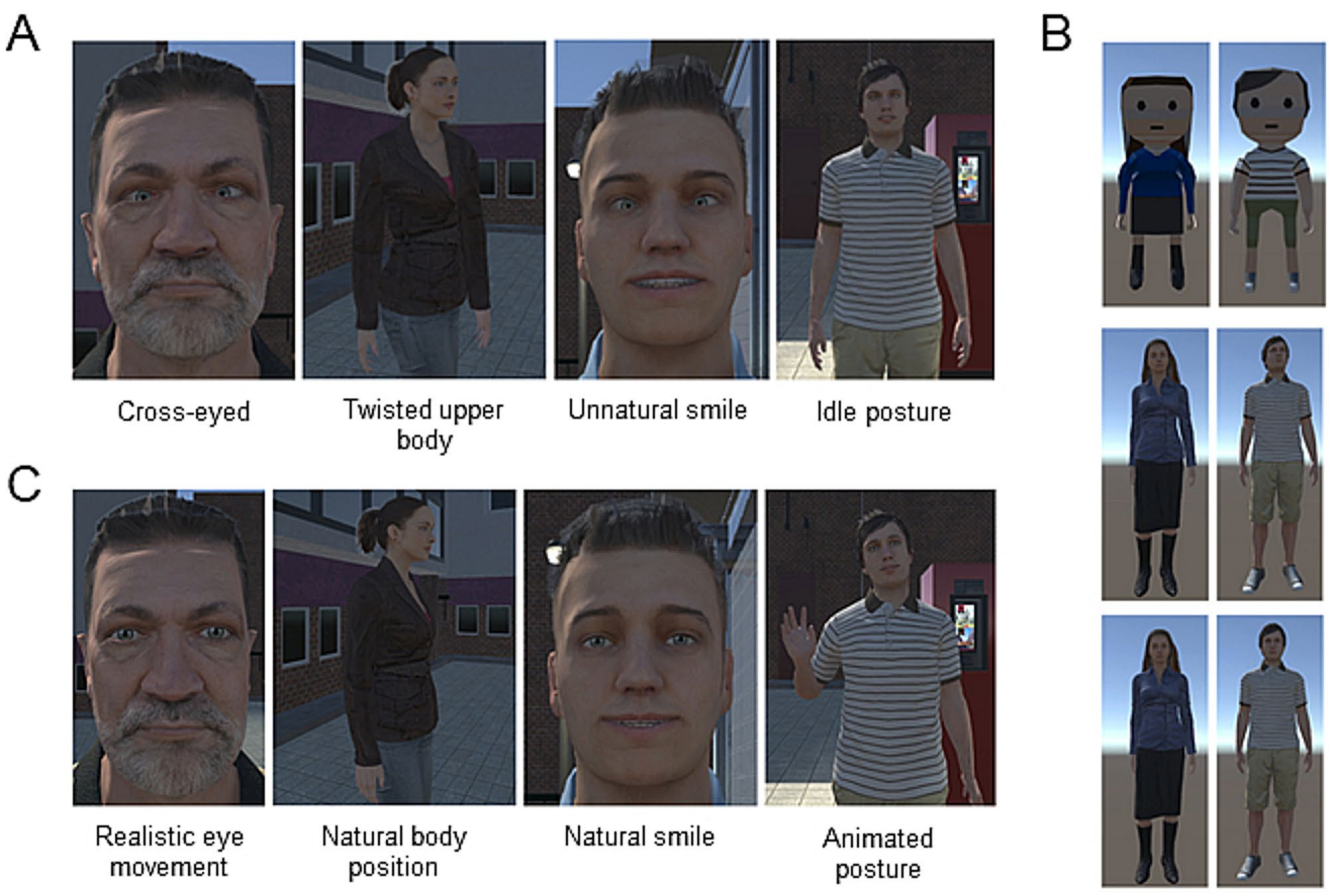
Examples from the different agent categories. **(A)** Four examples of the deviant features shown by the uncanny agents. These abnormalities in facial expression and movement pattern became particularly evident when the agents were approached. The cross-eyed gaze intensified, when the participant neared the agent, while the unnaturally twisted upper body occurred, for example, if the participant approached from the side. This was, because the agent followed the participant’s movement by moving its upper body. Their unnatural smile did not involve the eye region, but was restricted to the mouth. Apart from the rotation of the upper body, the uncanny agents’ body remained in an idle posture during the interaction. The displayed agents are from the Microsoft Rocketbox Avatar Library^59^. **(B)** Examples of the cartoon agents (first row) and matched examples of the uncanny agents (second row) and the realistic agents (third row). The agents displayed in the second and third row are from the Microsoft Rocketbox Avatar Library^59^. **(C)** Four examples of the improved features of the realistic agents. The abnormalities in facial expression and movement patterns of the uncanny agents were reduced during the social interaction to increase acceptance. The improved virtual agents displayed are based on the agents provided by the Microsoft Rocketbox Avatar Library^59^.

The VE consisted of the bus stop scenario used by Keller et al.^7^ around which the 10 agents of the respective category were distributed. The uncanny agent category comprised the same agents (6f, 4 m) from the *Microsoft Rocketbox Avatar Library*^59^. We hypothesized, that the agents in this condition were perceived as uncanny due to slightly aberrant and inconsistent features (see Fig. 4A for examples). The cartoon agents were created using the software *Blender* (version 3.6). They were designed similar to the human-like agents, but we avoided an excessive level of realism to keep them out of the realm of the uncanny valley. Ten different cartoon agents were created using the male and female template. Each cartoon character resembled one of the human-like agents in terms of skin coloration, hairstyle, hair color, face shape, clothing, and accessories like necklaces and watches (see Fig. 4B), and was placed in the same location of the VE as the corresponding agents in the other two conditions.

For the realistic agents the same models from the *Microsoft Rocketbox Avatar Library* were used as in the uncanny category. However, we improved the realistic agents in order to make them less uncanny. For this, the following adjustments were made: (1) Realistic eye movement: To improve the eye movements and eye gaze behavior a *Realistic Eye Movements* Unity asset was used (Tore Knabe 2015, Realistic Eye Movements 29168. https://assetstore.unity.com). This asset was compatible with the Microsoft Rocketbox avatars and allowed the agents to move their eyes, heads, and eyelids in a lifelike way without being crossed-eyed. Previous work indicates that semi-photorealistic avatars benefit more from inferred than random gaze^60^. The *Look Target Controller* script provided by the Realistic Eye Movements asset allowed us to set points of interest and define details like the stare-back factor or the minimum and maximum looking time at the user. This was essential since previous work has shown the importance of the eyes and their impact on the character’s perceived eeriness^35,61^. (2) Natural body movements: The *C-sharp* script in Unity, which controlled the agents’ movements, was modified. In the realistic scenario, the agents did not rotate their upper bodies to track the participants’ movements. Instead, they tracked the participants’ movements using head and eye motions. This prevented them from ending up with a twisted upper body and helped them to act in a socially acceptable way, so they wouldn’t be rejected by the participants^62^. (3.) Natural smile: For each agent, a new smile animator in Unity was created. The goal of the new animators was to give the agents a convincing smile. This was achieved by the agents pulling up the corners of their mouths, opening their mouths slightly, and raising their cheeks and eyebrows. According to Ekman’s Facial Action Coding System these movements are important indicators of happiness^63^. Each smile animator was manually created and adjusted to match the agents’ appearance.

(4.) Animations: In addition to the smile condition an acknowledgment condition was created. This new condition activated acknowledgment animations such as waving and head nodding. As the preceding work has shown, viewers rated entities that show social behaviors such as head nodding and arm movement as more authentic^62^. The acknowledgment animations were triggered simultaneously with the smiling animation. We used animations provided by Mixamo (a free online tool that can rig custom 3D characters; https://www.mixamo.com) (see Fig. 4C for examples of realistic features).

### MAAST VR task

The participant was randomly assigned to one of three VR scenarios, which differed only with respect to the agent category (cartoon, uncanny, realistic) that had been developed in the Unity game engine (version 202.3.32f1). The virtual stimulus was displayed on a Meta Quest 2, which provided a dual LCD screen setup with 1832 × 1920 per eye resolution distributed over an approximate field of view of 110 degrees. During VR, the participant remained seated in front of a desk and navigated oneself in the virtual environment (VE) with the compatible controllers. The MAAST was originally developed by Keller et al.^7^ and is also described in detail there. After having completed the short VR tutorial, the participants entered the VE of a bus stop surrounded by a sidewalk, houses, and a street. Ten agents (6f/4m) were positioned around the sidewalk, either directly at the bus stop or in its vicinity. To complete the MAAST, the agents had to make each of the ten agents smile, which was accomplished by approaching the agents, looking into their eyes, and waiting until they smiled. The smiling animation for all cartoon agents, uncanny agents, and realistic agents was triggered when the following interaction requirements were met: (1) The participant stood three or less meters in front of the agent. (2) The participant positioned themselves within the agent’s frontal field of vision, spanning 120 degrees. (3) The participant looked at the agent. (4) The individual time to smile of the agent was over. When interaction requirements one, two, and three were fulfilled, the timer of the time to smile began. When the timer finished counting down, the smile was initiated. Initially, all agents had a neutral expression, yet differed in their general appearance (clothes, age, sex etc.) and in the time to smile (time to smile averaged at about 15 s, ranging from 10s to 25s). Once the smile was provoked, the agent remained smiling, making it easier for the participant to keep track of the task progress. For the realistic agents, the acknowledgment animations were triggered when all interaction requirements were met. After the acknowledgment animations were performed, the realistic agents returned to their idle animations.

### sIgA sampling and analysis

The saliva samples were collected in 2mL microcentrifuge tubes by passive drool. The experimenter measured the time it took the participant to fill up the tube. Subsequently, the samples were weighed and frozen at -20 °C. After completion of data collection, samples were anonymized and sent to an external laboratory (*MVZ Volkmann Laboratory*, Karlsruhe, Germany), where they were analyzed with Nephelometry (mg/dL) with an accredited analysis protocol. The resulting sIgA concentration was converted from milligrams per deciliter to micrograms per milliliter (µg/mL) and then multiplied by the flow rate, which is the saliva volume (mL) divided by the time of sample collection (min) [For this purpose, the weight of the sample (mg), assuming that the weight of 1 mL saliva corresponds to that of 1 mL water, was converted 1:1 into milliliters.]. The resulting sIgA secretion rate (µg/min) was then used in the statistical analysis. Based on the secretion of the baseline and the post-VR samples we also calculated the ΔsIgA, which is the difference of post-VR minus baseline and thus indicates the change in sIgA secretion rate during VR.

### Statistical methods

We used the Kolmogorov-Smirnov-test as test of normality. Most variables showed a significant deviation from the Gaussian normal distribution (*p* < .05). Only the trait disgust and core disgust scores, the IPQ scales *Experienced Realism* and *Spatial Presence*, as well as the ratings of human-likeness and attractiveness of the realistic and uncanny agents did not significantly deviate from normality. Therefore, we decided to always use non-parametric tests for pairwise post-hoc comparisons and correlations (see below).

The influence of agent category group on the change in sIgA secretion rate was analyzed with a repeated-measures general linear model (GLM) that included the within-subject factor “sample” and a between-subject factor “group”. Significant effects in the GLM were further examined with non-parametric post-hoc tests, i.e., the Mann-Whitney-U-Test for independent data and the Wilcoxon test for paired data. Another repeated-measures GLM was used to examine the effect of “agent category” and “rating dimension” as well as “group” on the agent rating. For comparisons of the questionnaire scores of the three VR-groups we used the Kruskal-Wallis-test. Finally, Spearman rank correlations were used to test for linear associations between continuous variables. The statistical threshold was set to *p* < .05 (two-tailed) in all statistical tests.

## Supplementary Information

Below is the link to the electronic supplementary material.

Supplementary Material 1

## Data Availability

Data has been deposited in https://osf.io/7uq6r/. It can also be obtained directly from the corresponding author.
