# Supplementary material for: The uncanny valley effect and immune activation in virtual reality
Source: Sci Rep. 2025 Aug 19;15:30473. doi: 10.1038/s41598-025-15579-4 (PMC12365102; doi:10.1038/s41598-025-15579-4)
Supplement: Supplementary file 1 — Supplementary Material 1 [file 41598_2025_15579_MOESM1_ESM.pdf]

Supplementary data

Table S1: Comparison of ratings of the different agent categories across the complete sample. We used a Wilcoxon-test for non-parametric data [table shows Z-values (p-values)]. Significant differences are marked with an asterisk and are highlighted in bold.

Comparison of ...	Realistic agents	Uncanny agents	Cartoon agents
(a) Human-likeness rating			
Realistic agents	-	**-2.48 (.013)***	**-7.04 (<.001)***
Uncanny agents	**-2.48 (.013)***	-	**-6.91 (<.001)***
Cartoon agens	**-7.04 (<.001)***	**-6.91 (<.001)***	-
(b) Attractiveness rating			
Realistic agents	-	**-3.21 (.001)***	**-6.91 (<.001)***
Uncanny agents	**-3.21 (.001)***	-	**-6.60 (<.001)***
Cartoon agens	**-6.91 (<.001)***	**-6.60 (<.001)***	-
(c) Eeriness rating			
Realistic agents	-	**-4.22 (<.001)***	-.51 (.609)
Uncanny agents	**-4.22 (<.001)***	-	**-2.34 (.019)***
Cartoon agens	-.51 (.609)	**-2.34 (.019)***	-

Table S2: Pairwise comparisons of the ratings of human-likeness between the different agent groups. We used a Mann-Whitney-U-test for non-parametric data [table shows Z-values (p-values)]. Significant differences are marked with an asterisk and are highlighted in bold.

Comparison of ...	Realistic group	Uncanny group	Cartoon group
(d) Human-likeness of realistic agents			
Realistic group	-	-1.72 (.086)	**-2.04 (.042)***
Uncanny group	-1.72 (.086)	-	-.43 (.668)
Cartoon group	**-2.04 (.042)***	-.43 (.668)	-
(e) Human-likeness of uncanny agents			
Realistic group	-	-1.05 (.295)	-.12 (.903)
Uncanny group	-1.05 (.295)	-	-1.25 (.210)
Cartoon group	-.12 (.903)	-1.25 (.210)	-
(f) Human-likeness of cartoon agents			
Realistic group	-	-.32 (.748)	**-4.72 (<.001)***
Uncanny group	-.32 (.748)	-	**-4.35 (<.001)***
Cartoon group	**-4.72 (<.001)***	**-4.35 (<.001)***	-

Table S3: Pairwise comparisons of the ratings of attractiveness between the different agent groups. We used a Mann-Whitney-U-test for non-parametric data [table shows Z-values (p-values)]. Significant differences are marked with an asterisk and are highlighted in bold.

Comparison of ...	Realistic group	Uncanny group	Cartoon group
*(a) **Attractiveness of realistic agents***			
Realistic group	-	-.62 (.534)	-.83 (.407)
Uncanny group	-.62 (.534)	-	-.86 (.391)
Cartoon group	-.83 (.407)	-.86 (.391)	-
*(b) **Attractiveness of uncanny agents***			
Realistic group	-	-.16 (.874)	-1.91 (.056)
Uncanny group	-.16 (.874)	-	**-2.06 (.040)***
Cartoon group	-1.91 (.056)	**-2.06 (.040)***	-
*(c) **Attractiveness of cartoon agents***			
Realistic group	-	-.08 (.940)	**-2.11 (.035)***
Uncanny group	-.08 (.940)	-	-1.94 (.052)
Cartoon group	**-2.11 (.035)***	-1.94 (.052)	-

Table S4: Pairwise comparisons of the ratings of eeriness between the different agent groups. We used a Mann-Whitney-U-test for non-parametric data [table shows Z-values (p-values)]. Significant differences are marked with an asterisk and are highlighted in bold.

Comparison of ...	Realistic group	Uncanny group	Cartoon group
*(a) **Eeriness of realistic agents***			
Realistic group	-	-.13 (.893)	-.09 (.932)
Uncanny group	-.13 (.893)	-	-.24 (.809)
Cartoon group	-.09 (.932)	-.24 (.809)	-
*(b) **Eeriness of uncanny agents***			
Realistic group	-	-1.04 (.300)	-1.21 (.228)
Uncanny group	-1.04 (.300)	-	-.52 (.605)
Cartoon group	-1.21 (.228)	-.52 (.605)	-
*(c) **Eeriness of cartoon agents***			
Realistic group	-	-.81 (.420)	-.81 (.420)
Uncanny group	-.81 (.420)	-	-.34 (.732)
Cartoon group	-.81 (.420)	-.34 (.732)	-
